# Model-assisted analysis of the peach pedicel–fruit system suggests regulation of sugar uptake and a water-saving strategy

**DOI:** 10.1093/jxb/eraa103

**Published:** 2020-05-18

**Authors:** Dario Constantinescu, Gilles Vercambre, Michel Génard

**Affiliations:** 1 UR 1115 PSH, INRAE, Avignon Cedex 9, France; 2 University of Essex, UK

**Keywords:** Apoplast, fruit, model, pedicel, sugar, symplast, uptake, water

## Abstract

We develop a model based on the biophysical representation of water and sugar flows between the pedicel, fruit xylem and phloem, and the fruit apoplast and symplast in order to identify diurnal patterns of transport in the pedicel–fruit system of peach. The model predicts that during the night water is mainly imported to the fruit through the xylem, and that fruit phloem–xylem transfer of water allows sugar concentrations in the phloem to be higher in the fruit than in the pedicel. This results in relatively high sugar transport to the fruit apoplast, leading to relatively high sugar uptake by the fruit symplast despite low sugar concentrations in the pedicel. At midday, the model predicts a xylem backflow of water driven by a lower pressure potential in the xylem than in the fruit apoplast. In addition, fruit xylem-to-phloem transfer of water decreases the fruit phloem sugar concentration, resulting in moderate sugar uptake by the fruit symplast, despite the high sugar concentration in the pedicel. Globally, the predicted fruit xylem–phloem water transfers buffer the sugar concentrations in the fruit phloem and apoplast, leading to a diurnally regulated uptake of sugar. A possible fruit xylem-to-apoplast recirculation of water through the fruit phloem reduces water lost by xylem backflow at midday.

## Teaser

Regulation of water flow in the pedicel–fruit system may generate a diurnally regulated uptake of sugar and prevent water loss by a system of fruit xylem-to-phloem and phloem-to-apoplast recirculation.

## Introduction

Inflow of water to fruit is determined by transport that occurs in the vascular system of the pedicel and it varies with plant water status during the day (Tromp, 1984; Guichard *et al.*, 2005; Matthews and Shackel, 2005; Morandi and Grappadelli, 2008). Water is lost from fruit through transpiration, which can contribute significantly to diurnal fruit contraction (Clearwater *et al.*, 2012; Brüggenwirth *et al.*, 2016). Backflow through the xylem may also contribute to water loss from fruit, driven by a higher pressure potential in the fruit than in the plant xylem (Zhang and Keller, 2017). Backflows have been observed experimentally in many species at different stages of fruit growth (Lang and Thorpe, 1989; Huguet *et al.*, 1997; Keller *et al.*, 2006; Carlomagno *et al.*, 2018). Although many studies have been conducted on fruit water flows at both hourly and diurnal scales, measures of fruit water balance have always been indirect and this can lead to systematic errors (Fishman *et al.*, 2001). Recently, non-invasive methods such as positron emission tomography (PET) and magnetic resonance imaging (MRI) have been used to determine fruit and pedicel water relations. These techniques have also been used to assess the contribution of the xylem to fruit inflows in the late stages of growth, and to better understand the decline in xylem functionality in grape and tomato (Windt *et al.*, 2009; Knipfer *et al*., 2015; Van de Wal *et al.*, 2017).

Fruit dry matter accumulation mainly results from sugar transport processes. According to Munch’s theory, sugars are initially transferred to the fruit through the pedicel phloem by mass flow that is driven by higher pressure potential in the plant than in the fruit (Thompson and Holbrook, 2003). Translocation of sugars from the fruit sieve elements into the fruit cells happens via either symplastic or apoplastic pathways. Symplastic transport consists of mass flow through plasmodesmata from the sieve element to the fruit cell cytoplasm. In apoplastic transport, sugars are first transported from the phloem sieve element to the apoplast surrounding the fruit cell through a combination of diffusion (Patrick and Offler, 1996) and active transport (Lalonde *et al.*, 2003; Zhang *et al.*, 2004; Lemoine *et al.*, 2013), and then they are transferred into the cell cytoplasm through active transport (Ruan and Patrick, 1995; Manning *et al.*, 2001). Transport of sugars in the fruit leads to changes in water flows, since sugar accumulation alters the water potential difference between the plant and the fruit (Zhu *et al.*, 2019). This significant interconnection between the flows of water and sugar has been highlighted by Keller *et al.* (2015), who have proposed a conceptual model of water and sugar movements in grape berries during ripening. According to this model, at late ripening the water inflow from the fruit phloem to the fruit cell apoplast exceeds the transpiration demand and sustains both fruit growth and solute accumulation. This water inflow via the phloem increases the pressure potential of the apoplast surrounding the fruit cell, and it becomes higher than the pedicel xylem pressure potential. Thus, part of the water coming in from the phloem evaporates by transpiration, part is moved from the apoplast surrounding the fruit cell to the cell cytoplasm in order to sustain fruit growth, and another part is recirculated to the fruit xylem via the apoplast surrounding the fruit cell, with the latter having a higher pressure potential than the fruit xylem. The fruit growth model of Fishman and Génard (1998) is a mathematical tool developed to predict fruit growth by simulating the water and sugar flows that occur in the fruit xylem and phloem. In the extended versions proposed by Hall et al. (2013, 2017), a pedicel compartment and distinct apoplastic/symplastic pathways were added to the model.

The aim of this current study was to identify and describe the diurnal water flows and sugar transport that occur in the pedicel–fruit system by means of a simple mathematical tool. We have built a biophysical model of water and sugar flows across the system at an hourly scale, focusing on the pedicel, the fruit vascular system, and the cell apoplast. We mathematically describe water and sugar flows using the same paradigm as the existing fruit growth model, including the pedicel and the distinction between the apoplast and symplast that was proposed by Hall et al. (2013, 2017). We estimated the model parameters by calibration of the model in order to predict the diurnal variation in volume of a peach fruit under given conditions of crop load. The simulations highlighted different water and sugar transport patterns in the pedicel–fruit system.

## Material and methods

### Experimental treatments

The model was calibrated for the late-maturing peach (*Prunus persica* (L.) Batsch) cultivar ‘Suncrest’/GF 677. Measurements were performed on peach trees growing in the orchard of the INRA Avignon Centre, which received routine horticultural care. In 1994, the observed fruit-bearing shoots were thinned to a leaf-to-fruit ratio of 30, and in 1995 to either a leaf-to-fruit ratio of 5 (heavy crop load) or a leaf-to-fruit ratio of 30 (light crop load). Measurements were performed on fruits at the same growth stage in two different years: from 19–30 July 1994, and from 20–30 July 1995, both at 120–130 d after anthesis (DAA). Diurnal variations in fruit diameter were determined using linear variable differential transformer (LVDT) gauges, as described in Huguet *et al.* (1985). The number of fruits and the total number of days when they were measured are given in Table 1. The measured diameters, *D* (mm), were transformed into fresh weights *W* (g) using an empirical correlation for the ‘Suncrest’ cultivar: *W*=0.003*D*^2.58^ (Huguet *et al.*, 1997). Measurements were made on intact (control) fruits and ‘pedicel-girdled’ fruits, where the bearing shoot was girdled just below the pedicel to prevent the flow in the phloem from entering the pedicel. In addition, Fruits were detached at the beginning of the measurement period and suspended in the tree canopy at their original position in order to evaluate the water mass lost by transpiration. We considered that the dry weight was 10% of the mean fruit fresh weight measured for a given fruit during the day; this value was derived from data collected for ‘Suncrest’ fruit sampled from the same orchard. Climatic data collected at INRA weather stations located close to the experimental fields were used as model inputs.

**Table 1.** Number of fruits and total days for which the diameters were monitored for each fruit-load treatment growth condition

Treatment	Growing conditions	Number of fruits	Total days of monitoring
30 leaf-to-fruit, 1994	Control (intact)	3	31
	Girdled	5	29
	Detached	7	29
30 leaf-to-fruit, 1995	Control (intact)	1	9
	Girdled	2	6
	Detached	4	8
5 leaf-to-fruit, 1995	Control (intact)	1	8
	Girdled	2	5
	Detached	4	8

Treatment is the fruit load, expressed as the leaf-to-fruit ratio. Growing conditions were control (intact fruit), girdled pedicel, or detached fruit.

### Model description

**Fig. 1.**
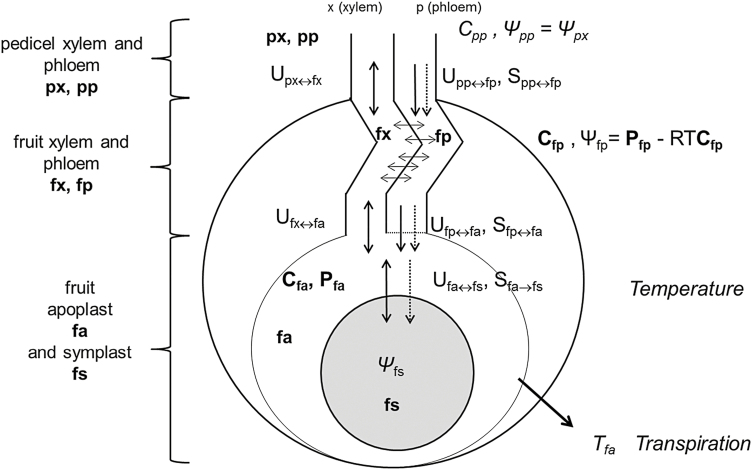
Conceptual model of the pedicel–fruit system in peach. We distinguish between the pedicel xylem and phloem (px, pp) and the fruit xylem and phloem (fx, fp), with the latter forming the fruit vascular system. The fruit cell apoplast (fa) surrounds the fruit cell symplast (fs). The external inputs of the model are represented in italics; the unknown variables of the linear system (eqns 18–21) are represented in bold. Arrows with solid lines represent water flows, and arrows with dotted lines represent sugar flows. The principal model hypotheses are expressed mathematically: the xylem and phloem have the same water potential, and the xylem pressure potential equals the xylem water potential.

The pedicel–fruit system is illustrated conceptually in Fig. 1. We consider the fruit as a big cell made of a symplast surrounded by an apoplast and a vascular system connected to the plant by the pedicel. The pedicel is divided into the pedicel xylem and phloem (px and pp, respectively). Water is transported from the pedicel to the fruit vascular system, which is composed of the fruit xylem and phloem (fx and fp, respectively); sugars are transported from the pedicel phloem to the fruit phloem by mass flow. We assume that the phloem and xylem water potentials are the same and that local water exchanges can maintain this equilibrium (Thompson and Holbrook, 2003; Hall and Minchin, 2013; Savage *et al.*, 2016). We also assume that sugars are the only osmotically active solutes. Solute concentrations are considered to be negligible both in the pedicel and fruit xylem, so that the xylem pressure potential equals the xylem water potential. The region where the fruit xylem and phloem terminate is represented as a system formed by the fruit cell apoplast (hereafter simplified to fruit apoplast, fa) connected to the vascular system and surrounding the fruit cell symplast (hereafter simplified to fruit symplast, fs) (as in the model of Hall *et al.*, 2017). A membrane separates the fruit phloem from the fruit apoplast, and we assume that there is no barrier for solutes between the fruit xylem and the fruit apoplast. Water is transported in the fruit symplast across a membrane and is lost through transpiration by the fruit apoplast.

The transport of sugars from the fruit phloem to the fruit symplast progressively shifts from the symplastic to the apoplastic pathway during fruit development (Damon *et al.*, 1988; Ruan and Patrick, 1995; Brown *et al.*, 1997; Moing *et al.*, 1997, Zhang *et al.*, 2006). We assume that the symplastic transport through plasmodesmata can be ignored during the last growth phase for which we collected experimental data (Zanon *et al.*, 2015). The apoplastic pathway comprises the following steps. (1) Sugars are transported from the fruit phloem to the fruit apoplast by both diffusion and active uptake. The transported sugar is mainly sucrose. (2) Sucrose is rapidly converted into hexoses by acid invertase in the fruit apoplast. (3) Hexoses are transported into the fruit symplast by active uptake.

We computed the water potential *Ψ*_w_ of each compartment as the sum of pressure potential *Ψ*_p_ and osmotic potential *Ψ*_π_ (all MPa):

$$\begin{array}{l} \Psi_{w}=\Psi_{p}+\Psi_{\pi } \end{array}$$ (1)

We computed the *Ψ*_π_ as the product of the gas constant R (g MPa K^−1^ mol^−1^), the temperature *T* (K), and sugar concentration *C* (mol g^−1^):

$$\begin{array}{l} \Psi_{\pi }=RTC \end{array}$$ (2)

We computed water flows (g h^−1^) following the equations used in the model of Hall *et al.* (2013). The water flow between pedicel phloem and fruit phloem (*U*_pp↔fp_), the flow between the pedicel xylem and fruit xylem (*U*_px↔fx_), and the flow between fruit xylem and fruit apoplast (*U*_fx↔fa_) are assumed to be directly proportional to the difference in pressure potential between the compartments, thus:

$$\begin{array}{l} U_{pp↔fp}=K_{pp↔fp}\left(\Psi_{p,pp}-\Psi_{p,fp}\right) \end{array}$$ (3)

$$\begin{array}{l} U_{px↔fx}=K_{px↔fx}\left(\Psi_{p,px}-\Psi_{p,fx}\right) \end{array}$$ (4)

$$\begin{array}{l} U_{fx↔fa}=k_{fx↔fa}A_{f}\left(\Psi_{p,fx}-\Psi_{p,fa}\right) \end{array}$$ (5)

Where *K*_pp↔fp_ and *K*_px↔fx_ (g MPa^−1^ h^−1^) are the conductances of the water flow paths between the pedicel phloem and fruit phloem, and between the pedicel xylem and fruit xylem, respectively, and *k*_fx↔fa_ (g MPa^−1^ h^−1^ cm^−2^) is the conductivity of the water flow path between the fruit xylem and fruit apoplast. We assume that the conductances of the water paths in the fruit between the fruit xylem and phloem and the fruit apoplast and between the fruit apoplast and the fruit symplast are proportional to the fruit surface area *A*_f_ (cm^2^), following the assumption of Fishman and Génard (1998).

The water flows (g h^−1^) that occur between the fruit phloem and the fruit apoplast (*U*_fp↔fa_) and between the fruit apoplast and the fruit symplast (*U*_fa↔fs_) are assumed to be directly proportional to the difference in water potential between the compartments, since we have assumed that water flows across membranes in this pathway. Hence:

$$\begin{array}{l} U_{fp↔fa}=k_{fp↔fa}A_{f}\left(\Psi_{w,fp}-\Psi_{w,fa}\right) \end{array}$$ (6)

$$\begin{array}{l} U_{fa↔fs}=k_{fa↔fs}A_{f}\left(\Psi_{w,fa}-\Psi_{w,fs}\right) \end{array}$$ (7)

Where *k*_fp↔fa_ and *k*_fa↔fs_ (g MPa^−1^ h^−1^ cm^−2^) are the conductivities of the water flow paths between the fruit phloem and fruit apoplast, and between the fruit apoplast and fruit symplast, respectively.

We assume that the sugar flow *S*_pp↔fp_ (g h^−1^) between the pedicel phloem and the fruit phloem is driven by mass flow:

$$\begin{array}{l} S_{pp↔fp}=M_{S}C_{pp}U_{pp↔fp} \end{array}$$ (8)

Where *M*_S_ (g mol^−1^) is the sucrose molar mass and *C*_pp_ (mol g^−1^) is the sucrose concentration in the pedicel phloem. *U*_pp↔fp_ is computed using eqn 3.

We assume that both diffusion and active uptake drive the sugar transport from the fruit phloem to the fruit apoplast and that the main transported sugar is sucrose. We consider that the diffusion process is driven by the difference between the fruit phloem and fruit apoplast sucrose concentrations, and that the fruit apoplast concentration is negligible compared to that of the fruit phloem. Ruan *et al.*, (1996) measured the sucrose concentration in tomato cell apoplast solutions and found a value of ~0.5 mM, which was indeed much lower than the fruit phloem concentrations estimated in our model. The rate of active sugar uptake from the fruit phloem to the fruit apoplast was then assumed to be proportional to the fruit phloem sugar concentration, for the sake of simplicity. In addition, we considered that both the diffusion and the active transport rates were directly proportional to the surface area of the exchange membrane between the fruit phloem and the fruit apoplast, which was assumed to be directly proportional to the fruit surface area.

We therefore computed the sugar flow *S*_fp→fa_ (g h^−1^) from the fruit phloem to the fruit apoplast as:

$$\begin{array}{l} S_{fp\to fa}=M_{S}v_{fp\to fa}A_{f}C_{fp} \end{array}$$ (9)

Where *v*_fp→fa_ (g cm^−2^ h^−1^) is the sugar transport coefficient considering both diffusion and active uptake, and *C*_fp_ (mol g^−1^) is the sucrose concentration in the fruit phloem.

We assume that the sugar flow from the fruit apoplast to the fruit symplast *S*^in^_fa→fs_ (g h^−1^) is directly proportional to the fruit apoplast sugar concentration *C*_fa_ (mol g^−1^) and to the fruit dry weight, as in Fishman and Génard (1998). Hence:

$$\begin{array}{l} S_{fa\to fs}^{in}=M_{H}v_{fa\to fs}^{in}C_{fa}DW \end{array}$$ (10)

Where *M*_H_ (g mol^−1^) is the hexoses molar mass, *v*^in^_fa→fs_ (h^−1^) is the coefficient of sugar transport from the fruit apoplast to the fruit symplast, and DW (g) is the fruit dry weight.

For the sake of simplicity, we assume that fruit respiration is a fraction *k*_Resp_ of the imported sugar, so we can compute the net sugar import *S*_fa→fs_ (g h^−1^) into the symplast as:

$$\begin{array}{l} S_{fa\to fs}=S_{fa\to fs}^{in}-S_{fs}^{out}=\;\left(1k_{Resp}\right)S_{fa\to fs}^{in} \end{array}$$ (11)

Where *S*^out^_fs_ (g h^−1^) is the sugar outflow by respiration.

The sugar net inflow *S*_fa→fs_ (g h^−1^) into the symplast is then expressed as:

$$\begin{array}{l} S_{fa\to fs}=M_{H}v_{fa\to fs}C_{fa}DW \end{array}$$ (12)

Where *v*_fa→fs_ is the coefficient of net sugar inflow into the symplast and can be seen as:

$$\begin{array}{l} v_{fa\to fs}=\;\left(1-k_{Resp}\right)v_{fa\to fs}^{in} \end{array}$$ (13)

### Model formulation

We assume that, at a given time of day, the system is at steady-state, and hence the pedicel xylem and phloem, the fruit xylem and phloem, and the fruit apoplast accumulate no sugar or water. This enables the formulation of four equations for mass conservation of water and sugars at any given time, as follows.

(1) The conservation of water flows through the xylem and phloem compartments between the pedicel and the fruit:

$$\begin{array}{l} U_{px↔fx}+U_{pp↔fp}=U_{fx↔fa}+U_{fp↔fa} \end{array}$$ (14)

(2) The conservation of water flows through the fruit xylem and phloem, the fruit apoplast and the fruit symplast:

$$\begin{array}{l} U_{fx↔fa}+U_{fp↔fa}=U_{fa↔fs}+T_{fa} \end{array}$$ (15)

Where *T*_fa_ is the fruit transpiration (g h^−1^).

(3) The conservation of sugars in the phloem between the pedicel and the fruit:

$$\begin{array}{l} S_{pp↔fp}=S_{fp\to fa} \end{array}$$ (16)

(4) The conservation of sugars in the fruit phloem, the fruit apoplast and the fruit symplast:

$$\begin{array}{l} S_{fp\to fa}=S_{fa\to fs} \end{array}$$ (17)

Based on the expressions for flows presented in this section, we can construct the following system:

$$\begin{array}{l} \begin{array}{ll} & K_{px↔fx}\left[\Psi_{w,pp}-\left(\Psi_{p,fp}-RTC_{fp}\right)\right]+\\ & K_{pp↔fp}\left[\left(\Psi_{w,pp}+RTC_{pp}\right)-\Psi_{p,fp}\right]-\\ & k_{fx↔fa}A_{f}\left[\left(\Psi_{p,fp}-RTC_{fp}\right)-\Psi_{p,fa}\right]-k_{fp↔fa}A_{f}+\\ & \left[\left(\Psi_{p,fp}-RTC_{fp}\right)-\left(\Psi_{p,fa}-RTC_{fa}\right)\right]\;=\;0 \end{array} \end{array}$$ (18)

$$\begin{array}{l} \begin{array}{ll} & k_{fx↔fa}A_{f}\left[\left(\Psi_{p,fp}RTC_{fp}\right)-\Psi_{p,fa}\right]\\ & +k_{fp↔fa}A_{f}\left[\left(\Psi_{p,fp}RTC_{fp}\right)-\left(\Psi_{p,fa}-RTC_{fa}\right)\right]-\\ & k_{fa↔fs}A_{f}\left[\left(\Psi_{p,fa}-RTC_{fa}\right)-\Psi_{w,fs}\right]-T_{fa}=\;0 \end{array} \end{array}$$ (19)

$$\begin{array}{l} M_{S}C_{pp}k_{pp↔fp}A_{f}\left[\left(\Psi_{w,pp}+RTC_{pp}\right)-\Psi_{p,fp}\right]-M_{S}v_{fp\to fa}A_{f}C_{fp}=0 \end{array}$$ (20)

$$\begin{array}{l} M_{S}v_{fp\to fa}A_{f}C_{fp}-M_{H}v_{fa\to fs}DWC_{fa}=0 \end{array}$$ (21)

Equations 18–21 compose a linear system with four unknown variables, which are presented in bold. We computed the algebraic expressions of these in terms of the other variables, i.e. model parameters and inputs, using the symbolic solver Sympy (Meurer *et al.*, 2017), and the solutions are shown in Supplementary Protocol S1 at *JXB* online. We could then compute water and sugar flows given the parameters and input values for each hour of the day. As already mentioned in the model description, we assume that local water exchanges allow the fruit xylem and the fruit phloem to be in equilibrium with regards to their water potential. Since there is conservation of water flow from the pedicel xylem to the fruit xylem, and then to the fruit apoplast (and likewise for the phloem), we compute the flow of water transfer between the fruit xylem to the fruit phloem as:

$$\begin{array}{l} U_{fx↔fp}=U_{px↔fx}-U_{fx↔fa} \end{array}$$ (22)

The model source code used here is available upon e-mail request to the corresponding author.

### Model inputs

In this study, we consider as model inputs all the variables representing system external conditions, i.e. pedicel water potential, pedicel phloem sugar concentration, fruit transpiration, fruit symplast water potential, and temperature. The pedicel water potential and the pedicel phloem sugar concentration were assumed to vary during the day (Hocking, 1980; Klages *et al.*, 2001). They were set to a constant value during the period 18.00–06.00 h and we assumed that they both followed a sinusoidal function (24 h period) between 06.00-18.00 h. The pedicel water potential was assumed to decrease until it reached a minimum value at 12.00 h, while we assumed that the pedicel phloem sugar concentration increased up to a maximum value at 12.00 h. Both variables then returned to their base value. The minimum and maximum values used for these inputs in the different treatments and years, and the time of day when the input variables had their extreme values, are shown in Table 2. The values of pedicel phloem water potential were set according to measurements of peach stem water potential obtained by Remorini and Massai (2003). The maximum values of sap sugar concentration were in the lower range for peach given by Jensen *et al.* (2013). The minimum values ranged within those given by Fishman and Génard (1998). We assumed that sugar concentrations in the pedicel phloem were lower in the light crop-load condition than in the heavy crop-load, as hypothesized by Fishman and Génard (1998). Estimation of the transpiration per unit area of the fruit surface was made by fitting a sinusoidal curve (24 h period) to the measured data obtained for the volume variation of detached fruits (see above), and this was then applied to the surface areas of the fruits considered for the model calibration. Fruit surface area *A*_f_ (cm^2^) was computed from fresh weight *W* (g) using the empirical relationship *A*_f_=6.049*W*^0.601^ (Fishman and Génard, 1998). We assumed that the symplast water potential was equal to the fruit water potential. Measurements on mango and tomato (Johnson *et al.*, 1992; Lechaudel, 2004) have suggested that fruit water potential is stable throughout the day; however, other measurements have shown a slight diurnal variability (McFayden *et al*., 1996; Morandi and Grappadelli, 2008). Hence, we assumed that the symplast water potential followed a sinusoidal behavior (24 h period) during the period 06.00–18:00 h, reaching a minimum at 12.00 h. We estimated the symplast water potential during the 18.00–06.00 h period, and hypothesized that the water potential at 12.00 h would 1.2-fold that of this value, as estimated by Morandi and Grappadelli (2008). We assumed that the fruit water potential changed according to treatment, year, and the conditions in which fruits were grown (control or girdled). Hereafter, ‘night’ refers to the period when the driving variables were constant, i.e. 18.00–06:00 h.

**Table 2.** Input variables of the model

Variable	Treatments	Minimum value	Maximum value
Pedicel phloem sugar concentration (g g^−1^)	30 leaf-to-fruit ratio, 1994 and 1995	0.12 (18.00–0.600 h)	0.28 (12.00 h)
	5 leaf-to-fruit ratio, 1995	0.08 (18.00–06.00 h)	0.24 (12.00 h)
Pedicel phloem water potential (MPa)	All	–1.3 (12.00 h)	–0.5 (18:00–06.00 h)
Fruit transpiration rate per area (g cm^−2^ h^−1^)	All	2.74×10^–4^ (05.00 h)	2.63×10^–3^ (17.00 h)

The times of day corresponding to the maximum and the minimum values are indicated.

### Model calibration and analysis of the responses of model variables to changing inputs

We estimated the model parameters in order to reproduce the mean variation in fruit fresh weight during a single day. We assumed that the biophysical parameters of the fruit did not vary during the measurement period. The system of eqns 18–21 was reduced for describing the girdled pedicel condition, imposing the absence of phloem flows (*U*_pp↔fp_=0 and *U*_fp↔fa_=0). A consequence of this system reduction was that sugar flows were equal to zero. This hypothesis agreed with measurements made by Génard *et al.* (2003), which showed that sugar accumulation immediately ceased in girdled fruits.

The simulated fruit fresh weight at a given hour, *h* (*W*_*h*_, g) was calculated as the accumulation of water and sugars in the fruit between a reference time *h*_0_ (W_ref,*h*0_) and *h*:

$$\begin{array}{l} W_{h}=W_{ref,h0}+S_{i=h0}^{h}\left[U_{fa↔fs,i}+S_{fa\to fs,i})\Delta i\right] \end{array}$$ (23)

Where *U*_fa↔fs,*i*_ and *S*_fa→fs,*i*_ are the water and sugar flows between the apoplast and the symplast at hour *i*, respectively. Δ*i* is the time step (1 h).

The objective function that we minimized was the root mean-squared error of the hourly predictions of fruit fresh weight (RMSE, g):

$$\begin{array}{l} RMSE=\sqrt{\frac{1}{N}\times \underset{h=1}{\overset{N}{\sum }}\;{\left(W_{h}-W_{obs,h}\right)}^{2}}\;\;\; \end{array}$$ (24)

Where *N* is the number of observed points during the measurement period, and *W*_*h*_ and *W*_obs,*h*_ are, respectively, the simulated and observed values of fruit fresh weight at hour *h*.

We aimed to minimize the RMSE index for both the simulations of the control (C) and the girdled (G) conditions, for one and two treatments in 1994 and 1995, respectively. To achieve this, we found the dominant solutions of a multi-objective optimization problem, minimizing both RMSE_C_ and RMSE_G_, where RMSE_C_ is the mean RMSE for the predicted fresh weight in the control for the different leaf-to-fruit ratios, and RMSE_G_ is the corresponding value in the girdled condition. We solved this problem using the multi-objective genetic algorithm NSGA-II (Deb *et al.*, 2002). Among the dominant solutions, we chose the one that resulted in the minimum mean value of (RMSE_C_+RMSE_G_). The parameters we estimated are shown in Table 3.

**Table 3.** Calibrated model parameters and brief descriptions

Parameter	Description	Value**	Units
*K*_px↔fx_	Conductance of the water path between the pedicel xylem and the fruit xylem	9.1×10^–1^	g h^−1^ MPa^−1^
*K*_pp↔fp_	Conductance of the water path between the pedicel phloem and the fruit phloem	3.3	g h^−1^ MPa^−1^
*k*_fx↔fa_	Conductivity and conductance of the water path between the fruit xylem and the fruit apoplast	1.1×10^–2^	g h^−1^ MPa^−1^ cm^−2^
*K*_fx↔fa_ *		7.4×10^–1^	g h^−1^ MPa^−1^
*k*_fp↔fa_	Conductivity and conductance of the water path between the fruit phloem and the fruit apoplast	1.1×10^–3^	g h^−1^ MPa^−1^ cm^−2^
*K*_fp↔fa_ *		7.6×10^–2^	g h^−1^ MPa^−1^
*k*_fa↔fs_	Conductivity and conductance of the water path between the fruit apoplast and the fruit symplast	9.1×10^–3^	g h^−1^ MPa^−1^ cm^−2^
*K*_fa↔fs_*		6.4×10^–1^	g h^−1^ MPa^−1^
*v*_fp↔fa_	Sugar transport rate between the fruit phloem and the fruit apoplast	5.0×10^–4^	g h^−1^ cm^−2^
*v*_fa→fs_	Fruit symplast sugar uptake rate	3.6×10^–2^	h^−1^
*Ψ*_fs_	Fruit symplast water potential (18.00–06.00 h)	–1.7 (C30_94)	MPa
		–1.7 (C30_95)	
		–1.3 (C5_95)	
		–0.89 (G30_94)	
		–1.1 (G30_95)	
		–0.91 (G5_95)	

C, control (intact) fruit; G, girdled fruit; 30_94, 30 leaf-to-fruit ratio, 1994; 5_95, 5 leaf-to-fruit ratio, 1995; 30_95, 30 leaf-to-fruit ratio, 1995.

* Conductances are given as the estimated value for the fruits grown in the 5 leaf-to-fruit treatment and in control conditions in 1995, which we took as a reference.

** The variability of the parameter estimations among the best solutions found using the genetic algorithm are listed in Supplementary Table S1.

In order to further verify the goodness of fit of our model predictions, we compared the contributions to the total water inflow of our predicted xylem and phloem inflows with the experimental observations of Morandi *et al.*, (2007) for peach fruits at stage III of growth. In addition, we also compared our predicted dry mass accumulation with the mean diurnal accumulation measured by Fishman and Génard (1998) on the same ‘Suncrest’ cultivar under heavy and light crop-load treatments.

In order to assess the dependence of our results on the model inputs, we analysed the responses of the outputs of the main model variables to different input levels of pedicel water potential and sugar concentration. This analysis is presented in Supplementary Table S1.1.

## Results

### Model calibration, and the diurnal contributions of xylem and phloem flows to the total water inflow

**Fig. 2.**
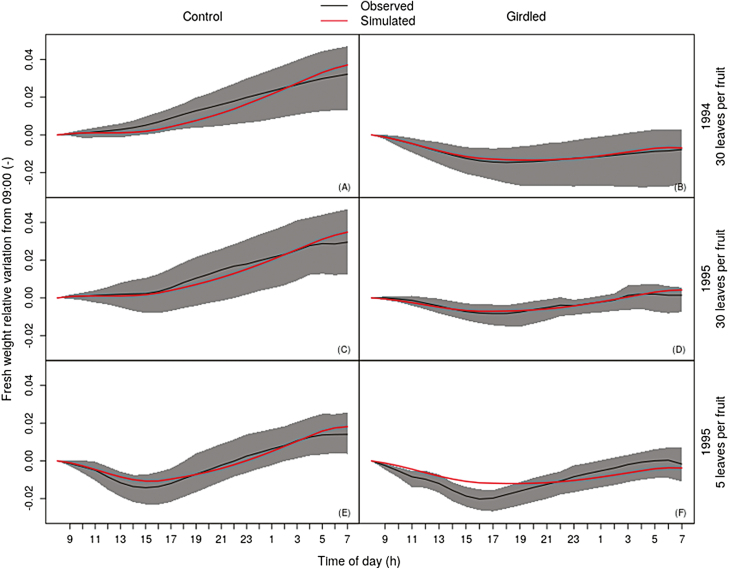
Calibration results for the model. The variations in fruit fresh weight relative to the value at 09.00 h are shown for the simulated (red lines) and observed (black lines) results. We calculated the relative variation as *W*_rel_=(*W*_*h*_ –*W*_ref_)/*W*_ref_, where *W*_*h*_ is the weight at a given hour, *h*, and *W*_ref_ is the weight at 09.00 h. The black lines are the mean values of the observations and the grey regions are delimited by the 5th and 95th percentiles, which were calculated based on the replications indicated in Table 1. (A, C, E) Control (intact) fruits, and (B, D, E) fruits with girdled pedicels, where the bearing shoot was girdled just before the fruit pedicel to prevent phloem flow from entering the pedicel. The years and crop-load conditions are indicated on the right.

The parameter values estimated through the model calibration are presented in Table 3, and Supplementary Table S1.1 shows their variability among the best solutions obtained in the calibration. The comparison between the predicted and the mean observed variations in diurnal fruit fresh weight are shown in Fig. 2, together with the 5th and 95th percentiles of the variation, which were computed from the replications of measurements summarized in Table 1. Globally, the variations in diurnal fruit mass observed in girdled conditions and the mass increase observed in control conditions in all treatments were reproduced well by the simulations. Furthermore, the differences between the behavior of fruit fresh mass among the crop-load treatments in control conditions were also reproduced well. However, the simulated girdled fruit fresh mass in the treatment with a leaf-to-fruit ratio of 5 had a smaller variation than the observed one. In both the 1994 and 1995 treatments with a leaf-to-fruit ratio of 30, the simulated diurnal contributions to the total water inflow were 20% and 80% for the phloem and xylem, respectively, while values in the treatment with a leaf-to-fruit ratio of 30 in 1995 were 29% and 71% for the phloem and xylem, respectively. These values agree with measurements made by Morandi *et al.* (2007) for stage III of peach fruit growth, namely 30% and 70% for the phloem and xylem contributions to the total water inflow, respectively. The simulated cumulative diurnal dry mass accumulation of the control fruits was 0.29 g d^−1^ in the treatment with a leaf-to-fruit ratio of 30 for both 1994 and 1995, and 0.12 g d^−1^ in the treatment with a leaf-to-fruit ratio of 5 in 1995. These values were similar to the mean diurnal dry mass accumulation measured by Fishman and Génard (1998) on the same cultivar, namely ~0.37 g d^−1^ for the light crop-load treatment and 0.09 g d^−1^ for the heavy crop-load treatment.

### The simulated fruit symplast sugar uptake is buffered compared to the variations in the pedicel phloem sugar concentration

**Fig. 3.**
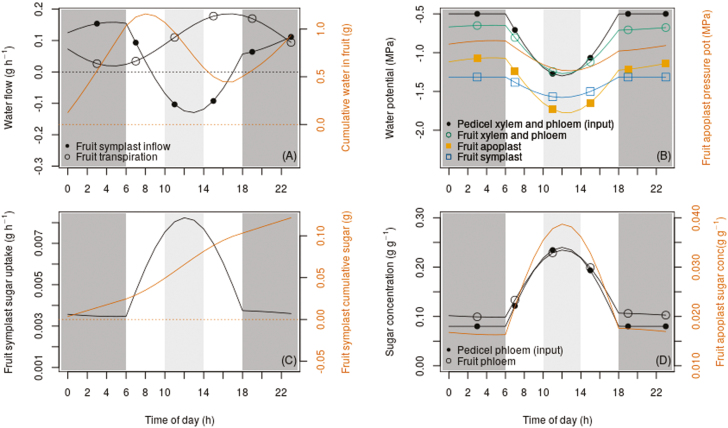
Simulated diurnal behavior of the variables related to fruit sugar transport and water exchanges in the pedicel–fruit system for a control (intact) fruit grown at a leaf-to-fruit ratio of 5. The night period (18.00–06.00 h) is shaded with dark grey and the midday period (10.00–14.00 h) is shaded with light grey. (A) Fruit symplast water inflow from the fruit apoplast, fruit transpiration, and cumulative water in the fruit (line with no symbols). (B) Input pedicel water potential, fruit xylem and phloem water potentials (assumed to be equal), fruit apoplast water potential, input fruit water potential, and fruit apoplast pressure potential (line with no symbols). (C) Fruit symplast sugar uptake and cumulative sugar stored in the fruit symplast. (D) Input pedicel sugar concentration, fruit phloem sugar concentration, and fruit apoplast sugar concentration (line with no symbols). Dotted lines indicate zero values of the variables.

We analysed the simulated diurnal behavior of the water and sugar flows in the treatment with a leaf-to-fruit ratio of 5, which we considered to be the most interesting pattern of transport. The input variables of this simulation are presented in the Methods and their values during the day are listed in Table 2. We compared the diurnal maximum relative variation of the symplast water inflow/outflow and of the sugar uptake with the diurnal maximum relative variation of the input variables related to water and sugar transport, i.e. the pedicel phloem water potential and sugar concentration. The maximum and minimum values of the diurnal symplast water inflow were 0.16 g h^−1^ and –0.13 g h^−1^, respectively (Fig. 3A), with a diurnal maximum relative variation of 1.8 (computed as |(max–min)/max|). This value was higher than relative variation of the maximum diurnal pedicel water potential input, which was 1.6 (computed with the same formula) (Fig. 3B). Therefore, variations in water potential input generated high variations in symplast water inflows and outflows. Examining this variation more closely, we calculated that the simulated symplast water inflow on average decreased by 0.06 g h^−1^ for every 0.2 MPa decrease in the pedicel water potential input. The minimum sugar uptake value was 0.0035 g h^−1^ and the maximum was 0.0082 g h^−1^ (Fig. 3C). The relative variation was 1.4 (computed as |max–min/min|), which was lower than the variation in the pedicel phloem sugar concentration input, the value of which was 2.0 (computed with the same formula). We obtained the same relative variation of 1.4 for both the fruit phloem and the fruit cell apoplast sugar concentrations (Fig. 3D). These results suggested that the fruit phloem and the fruit cell apoplast sugar concentrations together with the fruit symplast sugar uptake were buffered in response to the large variation in the pedicel phloem sugar concentration.

### In the middle part of the day, a xylem backflow of water is simulated, part of which is recirculated into the phloem and permits regulation of symplast sugar uptake

**Fig. 4.**
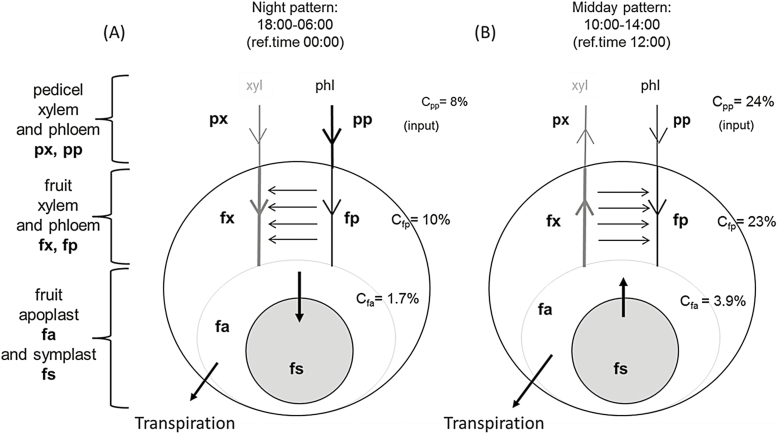
Water flow patterns in the pedicel–fruit system. (A) Water flows and sugar concentrations (*C*) during night (18.00–06.00 h) and (B) during the midday period (10.00–14.00 h). The arrows show the direction of flow. Grey arrows represent xylem flows and black arrows are phloem flows. Horizontal arrows represent water exchange between the fruit xylem and fruit phloem. The thicknesses of the lines qualitatively represents the magnitude of the flow. The sugar concentrations are the simulated values at the reference times of (A) 00.00 h and (B) 12.00 h.

We identified two main patterns of water and sugar flows, occurring during the night (18.00–06.00 h) and during the midday period (10.00–14.00 h) (Fig. 4).

**Fig. 5.**
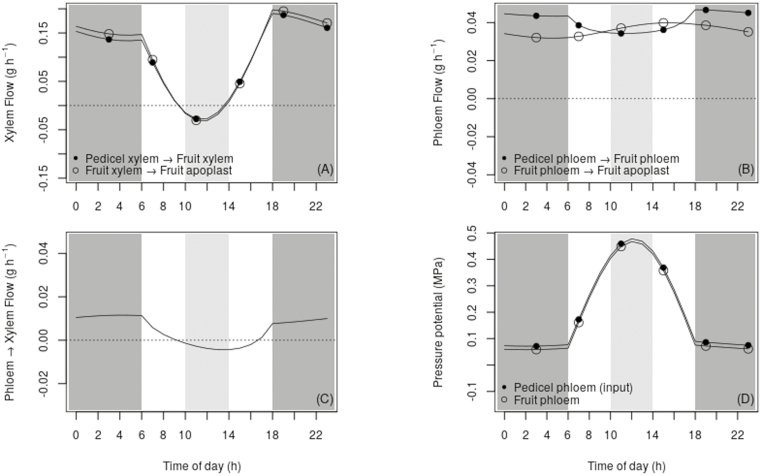
Simulated diurnal behavior of the variables related to water transport in the xylem and phloem pathways for a fruit grown at a leaf-to-fruit ratio of 5 under control conditions (i.e. intact fruit). The night period (18.00–06.00 h) is shaded with dark grey and the midday period (10.00–14.00 h) is shaded with light grey. (A) Water flow between the pedicel xylem and the fruit xylem, and from the fruit xylem to the fruit apoplast. (B) Water flow between the pedicel phloem and the fruit phloem, and from the fruit phloem to the fruit apoplast. (C) Water flow between the fruit xylem and the fruit phloem. (D) Input pressure potential in the pedicel phloem and the simulated fruit phloem pressure potential.

The model predicted that during the night, the fruit symplast imported water from the fruit apoplast (Fig. 3A). The main fruit water inflows were those of the xylem, which were higher than those of the phloem (Fig. 5A, B); however, the phloem inflows were not negligible in relation to those of the xylem. This predicted higher water flow from pedicel to fruit phloem than from fruit phloem to fruit cell apoplast (Fig. 5B) indicated that water was transported from the fruit phloem to the fruit xylem in the fruit vascular system (Fig. 5C, eqn 22). Specifically, ~20% of the water flow entering the fruit phloem was moved to the fruit xylem. Transpiration was the only fruit water outflow; this was low from 0.00–06.00 h and high at 18.00 h, sharply decreasing from 18:00–00.00 h (Fig. 3A). In summary, the model predicted that water was mainly imported via the pedicel xylem and lost by transpiration during the night. In addition, our simulations showed that part of the water entering the fruit via the phloem was transferred from the phloem to the fruit xylem, leading to higher sugar concentrations in the fruit phloem than in the pedicel phloem during this part of the day (Fig. 3D).

Our simulations show that during the midday period water was transferred from the fruit symplast to the fruit apoplast, and left the fruit apoplast by transpiration (Fig. 3A). In addition, we obtained a xylem water backflow from the fruit apoplast to the fruit xylem, and from the fruit xylem to the pedicel xylem. The magnitude of the backflow was comparable to that of the phloem flows (Fig. 5A, B). Simulated phloem flows were positive even though the magnitude of the water flow from the pedicel phloem to the fruit phloem decreased compared to the night-time value (Fig. 5B). In contrast to the night pattern, the model predicted a transfer of water from the fruit xylem to the fruit phloem, with the water flow from the pedicel phloem to the fruit phloem being lower than that from the fruit phloem to the fruit apoplast (Fig. 5B, C). In summary, a water backflow in the pedicel xylem and in the fruit xylem was predicted during the midday period. Moreover, it was predicted that part of the water flowing back from the fruit apoplast to the fruit xylem recirculated in a loop, passing from the fruit xylem to the fruit phloem, and from the fruit phloem back to the fruit apoplast. Since water was transferred from the fruit xylem to the fruit phloem, the increase in fruit phloem sugar concentration was buffered, reaching a slightly lower value than the pedicel phloem sugar concentration at midday. The slight variation of the fruit cell apoplast sugar concentration led to a regulated sugar uptake in the fruit symplast, generating an almost linear accumulation of sugar in the symplast (Fig. 3C).

By setting lower sugar concentrations in the pedicel phloem throughout the day, we simulated that water was mainly transferred from the fruit phloem to the fruit xylem. Conversely, for higher sugar concentrations in the pedicel phloem, water was mainly transferred from the fruit xylem to the fruit phloem (Supplementary Fig. S1.1). Moreover, the sugar accumulation in the fruit symplast remained almost linear throughout the day for both these input modifications (Supplementary Fig. S1.2).

The simulated water backflow was driven by higher pressure potential in the fruit apoplast than in the pedicel xylem. The simulated non-negligible mass flow of sugars was driven by slightly higher phloem pressure potential in the pedicel than in the fruit.

According to our simulation, the high and comparable xylem flows simulated in the pedicel xylem–fruit xylem pathway and in the fruit xylem–fruit apoplast pathway during the night (Fig. 5A) were explained by important pressure potential differences between the pedicel and the fruit xylem and between the fruit xylem and the fruit apoplast, respectively (Fig. 3B). During the midday period, the higher pressure potential of the fruit apoplast relative to that of the fruit xylem and the higher pressure potential of the fruit xylem relative to that of the pedicel xylem explained the non-negligible water backflows that were simulated (Fig. 5A). However, the backflow was lower than the flow of water entering the fruit during the night. Moreover, we estimated relatively high xylem conductances, *K*_px↔fx_ and *K*_fx↔fa_ (Table 3). In the analyses provided in Supplementary Fig. S1.1–2, we simulated that the xylem water backflow increased sensitively to decreasing values of the pedicel phloem water potential. Despite the low difference in pressure potential between the pedicel phloem and the fruit phloem (Fig. 5D), the mass flow from the former to the latter was not negligible (Fig. 5B). This flow was associated with a high phloem conductance, *K*_pp↔fp_ (Table 3). The water flow from fruit phloem to the fruit apoplast was constant during the whole day (Fig. 5B). This flow was guided by the high and constant difference in water potential between the fruit phloem and the fruit apoplast (Fig. 3A) and the relatively low conductance *K*_fp↔fa_ (Table 3).

## Discussion

Our simulations adequately reproduced the observed mean diurnal variations in the fresh weight of fruits grown under control and pedicel-girdled conditions in two different years and subject to two contrasting fruit-load treatments (Fig. 2). The simulation of the diurnal variation in the weight of girdled fruits grown in under a heavy crop load performed relative less well. This was probably due to the fact that the diurnal behavior of the pedicel water potential for this treatment was different to what we hypothesized, since we presumed that the water potential was the same for all the treatments in our model calibration. The contributions of the xylem and phloem inflows to the total water inflow in the control simulation agreed with the experimental observations of peaches at stage III of fruit growth reported by Morandi *et al.* (2007). Moreover, we simulated diurnal dry matter accumulation values that were similar to those measured by Fishman and Génard (1998) on the same cultivar for both the heavy and light crop-load treatments (Fig. 2A, C, E). Our analysis of the responses of the model variables to diurnal variations in inputs in the heavy crop-load treatment highlighted the fact that fruit phloem and fruit apoplast sugar concentrations were buffered in relation to the pedicel phloem sugar concentration given as the input. Furthermore, we computed a slight variation in the predicted symplast sugar uptake, which suggested that such a buffering effect could be a regulation strategy for sugar uptake (Fig. 3C). Analysis of the predicted diurnal variations in water and sugar transport and of their driving variables enabled the identification of two possible distinct water and sugar flow patterns that could explain this buffering effect and the consequent regulated uptake of sugar.

In the first pattern, during the night (Fig. 4A), the model predicted a transfer of water from fruit phloem to the fruit xylem that decreased the phloem pressure potential and maintained a high phloem sugar concentration (Fig. 3D). These conditions determined a high mass flow passing from the pedicel phloem to the fruit phloem and a high sugar diffusion from the fruit phloem to the fruit apoplast. Consequently, although the pedicel phloem sugar concentration given as the input was low, the predicted symplast sugar uptake was relatively high during this part of the day. Interestingly, we estimated a high value of conductance between the pedicel phloem and the fruit phloem, *K*_pp↔fp_, which compensated for the almost zero difference in pressure potential between the pedicel phloem and the fruit phloem (Fig. 5D). Indeed, the model predicted a non-negligible mass flow, and thus a realistic sugar uptake. This result was not surprising since very low differences of turgor pressure (i.e. pressure potential) have been experimentally determined in many herbaceous plants and trees, even though the biological reasons for non-negligible phloem mass flow in conditions of low pressure-potential differences between phloem compartments (and hence the reasons for high phloem conductance) are not yet well understood (Turgeon, 2010; Taiz *et al.*, 2015).

In the second pattern, in which occurred during the midday period (Fig. 4B), the model predicted a water backflow from the fruit cell apoplast to the xylem, due to lower pressure potentials in the xylem than in the apoplast (Fig. 5D). In contrast to the night-time simulations, during the midday period the model predicted a transfer of water from the fruit xylem to the fruit phloem. This transfer increased the fruit phloem pressure potential, thus limiting the mass flow and decreasing the fruit phloem sugar concentration, and hence reducing the diffusion of sugar from the fruit phloem to the fruit apoplast. As a consequence, the buffered sugar concentrations during the midday period resulted in a rate of symplast sugar uptake that was not much higher than that of the night period (Fig. 3C). The predicted sugar uptake was then lower than expected given a high sugar concentration in the pedicel phloem as an input. In the case of ripening grape berries, Keller *et al.* (2015), hypothesized that the water flowing from the phloem to the fruit cell apoplast (thus sustaining both fruit growth and solute accumulation) would be recirculated via xylem backflow since it exceeds the low transpiration demands. This flow of phloem water would be driven by a low osmotic potential in the fruit apoplast due to phloem sugar unloading. Furthermore, they hypothesized that the backflow could be driven by higher pressure potentials in the fruit apoplast than in the pedicel xylem. In our case, the predicted incoming water from the phloem did not exceed transpiration (Figs 3A, 5A). Nevertheless, our simulations showed that the hypotheses of Keller *et al.* (2015) are relevant and that water backflow in the fruit and in the pedicel xylem could be a common phenomenon due to the pressure potential differences that they considered. In addition, in our simulations the phloem water flow remained low during the midday period, with an absolute value comparable to the xylem water backflow (Fig. 5A, B). Interestingly, despite the high predicted water losses by backflow during this period, the predicted water flow from the fruit phloem to the fruit apoplast kept the same magnitude as that of the night period. This level of flow was driven by an apoplast water potential that was lower than that of the pedicel phloem and that was associated with low conductance between these two compartments, in agreement with the hypotheses of Keller *et al.* (2015). We predicted water recirculation from the fruit xylem to the fruit phloem during the midday period. Together with the xylem backflow, this recirculation could lead to favorable conditions for water flow from the fruit phloem to the fruit apoplast. This could be a strategy to reduce the water loss due to xylem backflow during the midday period. Furthermore, the relatively high conductance between the fruit xylem and fruit apoplast (*K*_fx↔fa_) suggested that for the peach, fruit xylem conductance may remain high during the late growth stage, which is in contrast to what happens in other fruit species where a decrease in xylem conductance occurs near ripening (Lang and Düring, 1991; Dichio *et al.*, 2002).

Our analysis of the responses of the model variables to different levels of inputs (Supplementary Fig. S1) agreed with what we observed in the simulations considered above. According to our simulations, when lower sugar concentrations were set in the pedicel phloem throughout the day, water was mainly transferred from the fruit phloem to the fruit xylem (Fig. 5C). This transfer would reduce the fruit phloem pressure potential and facilitate mass flow. With higher concentrations in the pedicel phloem, the simulation showed that water was mainly transferred from xylem to phloem. This transfer would facilitate water flow from the fruit phloem to the fruit apoplast and prevent water loss. In addition, this analysis allowed us to confirm that, in our system representation, the xylem water backflow strongly depended on the variations in input for pedicel water potential.

The model that we have developed is a simple biophysical representation of the pedicel–fruit system, and some of our hypotheses merit further discussion. First, the parameters used to build our model drive complex physiological processes and are, therefore, difficult to measure experimentally. Lacking literature values for these parameters, we estimated them through model calibration. Techniques such as positron emission tomography (PET) and magnetic resonance imaging (MRI) are promising tools to measure fluxes, and hence to estimate ratios between the xylem and phloem conductance parameters. Second, in our representation of the system, we defined a sequence of steady states in which the xylem and phloem water potentials equal each other (eqns 18–21). The assumption of different values for these water potentials could modify the computed water flow patterns, and hence our interpretation of the results. However, this would require the biophysical description of the lateral xylem–phloem flow that results from the differences in water potential between the fruit xylem and phloem. This would necessitate the estimation of an additional lateral conductivity parameter, which is difficult to obtain experimentally (Savage *et al.*, 2016). Third, we considered that only sucrose is transported in the pedicel phloem. Indeed, most of the sugar in peach sap is composed of sucrose, with only 35% being sorbitol (Desnoues *et al.*, 2018). Nevertheless, our model probably underestimates the phloem pressure potential because the sorbitol molar mass is nearly half that of sucrose. Moreover, the simulated xylem water backflow from the fruit apoplast to the pedicel xylem across the fruit vascular system would generate a sugar mass flow following the same pathway; these sugars would probably be reloaded by the phloem (Lang and Thorpe, 1989). However, this sugar transport from the fruit apoplast to the fruit phloem would only have an effect around midday, with a negligible contribution to the cumulative fruit sugar uptake. Furthermore, we assumed that the fruit apoplast sucrose concentration was negligible. Surprisingly, experimental measurements for this are lacking in the published literature. However, the sugar transport from the fruit phloem to the fruit apoplast is a combination of both diffusion and active uptake. The latter is driven only by the fruit phloem sucrose concentration and not by the sucrose concentration in the fruit apoplast. Finally, the sucrose concentration in the fruit cell apoplast is most likely much lower than that of the fruit phloem since there is high activity of sucrose transformation into hexoses by acid invertase. Overall, therefore, consideration of the apoplast sucrose concentration would probably not alter the conclusions about the translocation of sugars in the whole system.

## Conclusions and perspectives

In this study, we built a process-based model that allowed a description of the water transfers and sugar transport that occur in the pedicel–fruit system. We calibrated the model and our simulations gave good reproductions of diurnal variations in fresh weight for fruits naturally attached to the plant and for fruits with girdled pedicels. The model predicted that water transfers between the fruit xylem and the fruit phloem could generate a buffering effect on sugar concentrations in the fruit phloem and in the fruit apoplast during the day, which would result in a regulated uptake of sugar into the fruit and in a recirculation of water from the fruit phloem to the fruit apoplast during the middle part of the day. This suggested the presence of a regulation of sugar uptake and the prevention of water loss due to xylem backflow driven by the general water exchange pattern between the pedicel–fruit system compartments.

Looking ahead, our model could be a useful tool to identify water translocation and sugar accumulation strategies in other fruit. It may guide the physiological interpretation of the results of non-invasive methods used for analysing fruit water and sugar translocations, such as PET and MRI. In addition, with an improvement of the description of the lateral xylem–phloem water exchanges, the model simulations could further clarify patterns of water and sugar transport. Our model also describes an apoplastic step for sugar transport. In seeds, the apoplast contains high solute concentrations and is a key element in determining nutrient transport from the maternal seed tissues to the filial storage sites (Patrick and Offler, 2001). Our model therefore could also be adapted to help improve our understanding of the physiological mechanisms of water and sugar translocation in seeds.

## Supplementary data

Supplementary data are available at *JXB* online.

Protocol S1. Solutions for the linear system presented in eqns 18–21.

Table S1. Best solutions for calibrated model parameters, and their variability.

Fig. S1. Sensitivity of the model to different levels of inputs.

eraa103_suppl_Supplementary_File001

eraa103_suppl_Supplementary_File002

eraa103_suppl_Supplementary_File003
