# Supplementary material for: Model-assisted analysis of the peach pedicel–fruit system suggests regulation of sugar uptake and a water-saving strategy
Source: J Exp Bot. 2020 May 18;71(12):3463–74. doi: 10.1093/jxb/eraa103 (PMC7307860; doi:10.1093/jxb/eraa103)
Supplement: eraa103_suppl_Supplementary_File001 [file eraa103_suppl_supplementary_file001.pdf]

In this document, we provide the analytical solution of the system of equations presented in the section “*Model description and flow equations”* in the main text of this paper (Eq. 18-21). The equations of the system are the following (Eq. S1.1-S1.4). The unknown variables are marked in bold:

$K_{px\leftrightarrow fx}\times(\Psi_{w,pp} - (\boldsymbol{\Psi}_{\mathbf{p,fp}} - R\times T\times \mathbf{C}_{\mathbf{fp}})) + K_{pp\leftrightarrow fp}\times((\Psi_{w,pp} + R\times T\times C_{pp}) - \boldsymbol{\Psi}_{\mathbf{p,fp}}) +$

$- k_{fx\leftrightarrow fa}\times A_f\times((\boldsymbol{\Psi}_{\mathbf{p,fp}} - R\times T\times \mathbf{C}_{\mathbf{fp}}) - \boldsymbol{\Psi}_{\mathbf{p,fa}}) - k_{fp\leftrightarrow fa}\times A_f\times((\boldsymbol{\Psi}_{\mathbf{p,fp}} - R\times T\times \mathbf{C}_{\mathbf{fp}}) - (\boldsymbol{\Psi}_{\mathbf{p,fa}} - R\times T\times \mathbf{C}_{\mathbf{fa}})) = 0$ (S1.1)

$k_{fx\leftrightarrow fa}\times A_f\times((\boldsymbol{\Psi}_{\mathbf{p,fp}} - R\times T\times \mathbf{C}_{\mathbf{fp}}) - \boldsymbol{\Psi}_{\mathbf{p,fa}}) + k_{fp\leftrightarrow fa}\times A_f\times((\boldsymbol{\Psi}_{\mathbf{p,fp}} - R\times T\times \mathbf{C}_{\mathbf{fp}}) - (\boldsymbol{\Psi}_{\mathbf{p,fa}} - R\times T\times \mathbf{C}_{\mathbf{fa}})) +$

$- k_{fa\leftrightarrow fs}\times A_f\times((\boldsymbol{\Psi}_{\mathbf{p,fa}} - R\times T\times \mathbf{C}_{\mathbf{fa}}) - \Psi_{fs}) - T_{fa} = 0$ (S1.2)

$M_S\times C_{pp}\times k_{pp\leftrightarrow fp}\times A_f\times((\boldsymbol{\Psi}_{\mathbf{p},p} + R\times T\times C_{pp}) - \boldsymbol{\Psi}_{\mathbf{p,fp}}) - M_S\times v_{fp\rightarrow fa}\times A_f\times \mathbf{C}_{\mathbf{fp}} = 0$ (S1.3)

$M_S\times v_{fp\rightarrow fa}\times A_f\times \mathbf{C}_{\mathbf{fp}} - M_H\times v_{fa\rightarrow fs}\times DW\times \mathbf{C}_{\mathbf{fa}} = 0$ (S1.4)

The system analytical solutions are:

$\boldsymbol{\Psi}_{\mathbf{p,fp}} = (-DW\times M_H\times A_f\times v_{fp\rightarrow fa}\times v_{fa\rightarrow fs}\times(k_{fp\leftrightarrow fa} + k_{fx\leftrightarrow fa})\times(A_f\times k_{fa\leftrightarrow fs}\times\Psi_{fs} - T_{fa}) - DW\times M_H\times A_f\times v_{fp\rightarrow fa}\times v_{fa\rightarrow fs}\times(k_{fa\leftrightarrow fs} + k_{fp\leftrightarrow fa} + k_{fx\leftrightarrow fa})\times(R\times T\times C_{pp}\times K_{pp\leftrightarrow fp} + K_{pp\leftrightarrow fp}\times\Psi_{w,pp} + K_{px\leftrightarrow fx}\times\Psi_{w,pp}) + R\times T\times C_{pp}\times K_{pp\leftrightarrow fp}\times(R\times T\times C_{pp} + \Psi_{w,pp})\times(DW\times M_H\times A_f\times v_{fa\rightarrow fs}\times(k_{fp\leftrightarrow fa} + k_{fx\leftrightarrow fa})^2 - DW\times M_H\times v_{fa\rightarrow fs}\times(k_{fa\leftrightarrow fs} + k_{fp\leftrightarrow fa} + k_{fx\leftrightarrow fa})\times(A_f\times k_{fp\leftrightarrow fa} + A_f\times k_{fx\leftrightarrow fa} + K_{px\leftrightarrow fx}) + M_S\times A_f^2\times k_{fp\leftrightarrow fa}\times v_{fp\rightarrow fa}\times(k_{fa\leftrightarrow fs} + k_{fp\leftrightarrow fa} + k_{fx\leftrightarrow fa}) - M_S\times A_f^2\times v_{fp\rightarrow fa}\times(k_{fa\leftrightarrow fs} + k_{fp\leftrightarrow fa})\times(k_{fp\leftrightarrow fa} + k_{fx\leftrightarrow fa})))/(DW\times M_H\times R\times A_f\times T\times C_{pp}\times K_{pp\leftrightarrow fp}\times v_{fa\rightarrow fs}\times(k_{fp\leftrightarrow fa} + k_{fx\leftrightarrow fa})^2 - DW\times M_H\times R\times T\times C_{pp}\times K_{pp\leftrightarrow fp}\times v_{fa\rightarrow fs}\times(k_{fa\leftrightarrow fs} + k_{fp\leftrightarrow fa} + k_{fx\leftrightarrow fa})\times(A_f\times k_{fp\leftrightarrow fa} + A_f\times k_{fx\leftrightarrow fa} + K_{px\leftrightarrow fx}) + DW\times M_H\times A_f^2\times v_{fp\rightarrow fa}\times v_{fa\rightarrow fs}\times(k_{fp\leftrightarrow fa} + k_{fx\leftrightarrow fa})^2 - DW\times M_H\times A_f\times v_{fp\rightarrow fa}\times v_{fa\rightarrow fs}\times(k_{fa\leftrightarrow fs} + k_{fp\leftrightarrow fa} + k_{fx\leftrightarrow fa})\times(A_f\times k_{fp\leftrightarrow fa} + A_f\times k_{fx\leftrightarrow fa} + K_{pp\leftrightarrow fp} + K_{px\leftrightarrow fx}) + M_S\times R\times A_f^2\times T\times C_{pp}\times K_{pp\leftrightarrow fp}\times k_{fp\leftrightarrow fa}\times v_{fp\rightarrow fa}\times(k_{fa\leftrightarrow fs} + k_{fp\leftrightarrow fa} + k_{fx\leftrightarrow fa}) - M_S\times R\times A_f^2\times T\times C_{pp}\times K_{pp\leftrightarrow fp}\times v_{fp\rightarrow fa}\times(k_{fa\leftrightarrow fs} + k_{fp\leftrightarrow fa})\times(k_{fp\leftrightarrow fa} + k_{fx\leftrightarrow fa}))$ (S1.5)

$\mathbf{C}_{\mathbf{fp}} = DW\times M_H\times C_{pp}\times K_{pp\leftrightarrow fp}\times v_{fa\rightarrow fs}\times((k_{fp\leftrightarrow fa} + k_{fx\leftrightarrow fa})\times(A_f\times k_{fa\leftrightarrow fs}\times\Psi_{fs} - T_{fa}) + (A_f\times(k_{fp\leftrightarrow fa} + k_{fx\leftrightarrow fa})^2 - (k_{fa\leftrightarrow fs} + k_{fp\leftrightarrow fa} + k_{fx\leftrightarrow fa})\times(A_f\times k_{fp\leftrightarrow fa} + A_f\times k_{fx\leftrightarrow fa} + K_{pp\leftrightarrow fp} + K_{px\leftrightarrow fx}))\times(R\times T\times C_{pp} + \Psi_{w,pp}) + (k_{fa\leftrightarrow fs} + k_{fp\leftrightarrow fa} + k_{fx\leftrightarrow fa})\times(R\times T\times C_{pp}\times K_{pp\leftrightarrow fp} + K_{pp\leftrightarrow fp}\times\Psi_{w,pp} + K_{px\leftrightarrow fx}\times\Psi_{w,pp}))/(DW\times M_H\times R\times A_f\times T\times C_{pp}\times K_{pp\leftrightarrow fp}\times v_{fa\rightarrow fs}\times(k_{fp\leftrightarrow fa} + k_{fx\leftrightarrow fa})^2 - DW\times M_H\times R\times T\times C_{pp}\times K_{pp\leftrightarrow fp}\times v_{fa\rightarrow fs}\times(k_{fa\leftrightarrow fs} + k_{fp\leftrightarrow fa} + k_{fx\leftrightarrow fa})\times(A_f\times k_{fp\leftrightarrow fa} + A_f\times k_{fx\leftrightarrow fa} + K_{px\leftrightarrow fx}) + DW\times M_H\times A_f^2\times v_{fp\rightarrow fa}\times v_{fa\rightarrow fs}\times(k_{fp\leftrightarrow fa} + k_{fx\leftrightarrow fa})^2 - DW\times M_H\times A_f\times v_{fp\rightarrow fa}\times v_{fa\rightarrow fs}\times(k_{fa\leftrightarrow fs} + k_{fp\leftrightarrow fa} +$

$k_{fx\leftrightarrow fa})\times(A_f\times k_{fp\leftrightarrow fa} + A_f\times k_{fx\leftrightarrow fa} + K_{pp\leftrightarrow fp} + K_{px\leftrightarrow fx}) + M_S\times R\times A_f^2\times T\times C_{pp}\times K_{pp\leftrightarrow fp}\times k_{fp\leftrightarrow fa}\times v_{fp\rightarrow fa}\times(k_{fa\leftrightarrow fs} + k_{fp\leftrightarrow fa} + k_{fx\leftrightarrow fa}) - M_S\times R\times A_f^2\times T\times C_{pp}\times K_{pp\leftrightarrow fp}\times v_{fp\rightarrow fa}\times(k_{fa\leftrightarrow fs} + k_{fp\leftrightarrow fa})\times(k_{fp\leftrightarrow fa} + k_{fx\leftrightarrow fa}))$ (S1.6)

$\mathbf{\Psi_{p,fa}} = -(R\times A_f\times T\times C_{pp}\times K_{pp\leftrightarrow fp}\times(R\times T\times C_{pp} + \Psi_{w,pp})\times(DW\times M_H\times v_{fa\rightarrow fs}\times(k_{fp\leftrightarrow fa} + k_{fx\leftrightarrow fa})\times(A_f\times k_{fp\leftrightarrow fa} + A_f\times k_{fx\leftrightarrow fa} + K_{px\leftrightarrow fx}) - DW\times M_H\times v_{fa\rightarrow fs}\times(k_{fp\leftrightarrow fa} + k_{fx\leftrightarrow fa})\times(A_f\times k_{fp\leftrightarrow fa} + A_f\times k_{fx\leftrightarrow fa} + K_{pp\leftrightarrow fp} + K_{px\leftrightarrow fx}) - M_S\times A_f^2\times k_{fp\leftrightarrow fa}\times v_{fp\rightarrow fa}\times(k_{fp\leftrightarrow fa} + k_{fx\leftrightarrow fa}) + M_S\times A_f\times v_{fp\rightarrow fa}\times(k_{fa\leftrightarrow fs} + k_{fp\leftrightarrow fa})\times(A_f\times k_{fp\leftrightarrow fa} + A_f\times k_{fx\leftrightarrow fa} + K_{pp\leftrightarrow fp} + K_{px\leftrightarrow fx})) + A_f\times(R\times T\times C_{pp}\times K_{pp\leftrightarrow fp} + K_{pp\leftrightarrow fp}\times\Psi_{w,pp} + K_{px\leftrightarrow fx}\times\Psi_{w,pp})\times(DW\times M_H\times R\times T\times C_{pp}\times K_{pp\leftrightarrow fp}\times v_{fa\rightarrow fs}\times(k_{fp\leftrightarrow fa} + k_{fx\leftrightarrow fa}) + DW\times M_H\times A_f\times v_{fp\rightarrow fa}\times v_{fa\rightarrow fs}\times(k_{fp\leftrightarrow fa} + k_{fx\leftrightarrow fa}) - M_S\times R\times A_f\times T\times C_{pp}\times K_{pp\leftrightarrow fp}\times v_{fp\rightarrow fa}\times(k_{fa\leftrightarrow fs} + k_{fp\leftrightarrow fa})) + (A_f\times k_{fa\leftrightarrow fs}\times\Psi_{fs} - T_{fa})\times(DW\times M_H\times R\times T\times C_{pp}\times K_{pp\leftrightarrow fp}\times v_{fa\rightarrow fs}\times(A_f\times k_{fp\leftrightarrow fa} + A_f\times k_{fx\leftrightarrow fa} + K_{px\leftrightarrow fx}) + DW\times M_H\times A_f\times v_{fp\rightarrow fa}\times v_{fa\rightarrow fs}\times(A_f\times k_{fp\leftrightarrow fa} + A_f\times k_{fx\leftrightarrow fa} + K_{pp\leftrightarrow fp} + K_{px\leftrightarrow fx}) - M_S\times R\times A_f^2\times T\times C_{pp}\times K_{pp\leftrightarrow fp}\times k_{fp\leftrightarrow fa}\times v_{fp\rightarrow fa}))/(A_f\times(DW\times M_H\times R\times A_f\times T\times C_{pp}\times K_{pp\leftrightarrow fp}\times v_{fa\rightarrow fs}\times(k_{fp\leftrightarrow fa} + k_{fx\leftrightarrow fa})^2 - DW\times M_H\times R\times T\times C_{pp}\times K_{pp\leftrightarrow fp}\times v_{fa\rightarrow fs}\times(k_{fa\leftrightarrow fs} + k_{fp\leftrightarrow fa} + k_{fx\leftrightarrow fa})\times(A_f\times k_{fp\leftrightarrow fa} + A_f\times k_{fx\leftrightarrow fa} + K_{px\leftrightarrow fx}) + DW\times M_H\times A_f$ (S1.7)

$\mathbf{C_{fa}} = M_S\times A_f\times C_{pp}\times K_{pp\leftrightarrow fp}\times v_{fp\rightarrow fa}\times((k_{fp\leftrightarrow fa} + k_{fx\leftrightarrow fa})\times(A_f\times k_{fa\leftrightarrow fs}\times\Psi_{fs} - T_{fa}) + (A_f\times(k_{fp\leftrightarrow fa} + k_{fx\leftrightarrow fa})^2 - (k_{fa\leftrightarrow fs} + k_{fp\leftrightarrow fa} + k_{fx\leftrightarrow fa})\times(A_f\times k_{fp\leftrightarrow fa} + A_f\times k_{fx\leftrightarrow fa} + K_{pp\leftrightarrow fp} + K_{px\leftrightarrow fx}))\times(R\times T\times C_{pp} + \Psi_{w,pp}) + (k_{fa\leftrightarrow fs} + k_{fp\leftrightarrow fa} + k_{fx\leftrightarrow fa})\times(R\times T\times C_{pp}\times K_{pp\leftrightarrow fp} + K_{pp\leftrightarrow fp}\times\Psi_{w,pp} + K_{px\leftrightarrow fx}\times\Psi_{w,pp}))/(DW\times M_H\times R\times A_f\times T\times C_{pp}\times K_{pp\leftrightarrow fp}\times v_{fa\rightarrow fs}\times(k_{fp\leftrightarrow fa} + k_{fx\leftrightarrow fa})^2 - DW\times M_H\times R\times T\times C_{pp}\times K_{pp\leftrightarrow fp}\times v_{fa\rightarrow fs}\times(k_{fa\leftrightarrow fs} + k_{fp\leftrightarrow fa} + k_{fx\leftrightarrow fa})\times(A_f\times k_{fp\leftrightarrow fa} + A_f\times k_{fx\leftrightarrow fa} + K_{px\leftrightarrow fx}) + DW\times M_H\times A_f^2\times v_{fp\rightarrow fa}\times v_{fa\rightarrow fs}\times(k_{fp\leftrightarrow fa} + k_{fx\leftrightarrow fa})^2 - DW\times M_H\times A_f\times v_{fp\rightarrow fa}\times v_{fa\rightarrow fs}\times(k_{fa\leftrightarrow fs} + k_{fp\leftrightarrow fa} + k_{fx\leftrightarrow fa})\times(A_f\times k_{fp\leftrightarrow fa} + A_f\times k_{fx\leftrightarrow fa} + K_{pp\leftrightarrow fp} + K_{px\leftrightarrow fx}) + M_S\times R\times A_f^2\times T\times C_{pp}\times K_{pp\leftrightarrow fp}\times k_{fp\leftrightarrow fa}\times v_{fp\rightarrow fa}\times(k_{fa\leftrightarrow fs} + k_{fp\leftrightarrow fa} + k_{fx\leftrightarrow fa}) - M_S\times R\times A_f^2\times T\times C_{pp}\times K_{pp\leftrightarrow fp}\times v_{fp\rightarrow fa}\times(k_{fa\leftrightarrow fs} + k_{fp\leftrightarrow fa})\times(k_{fp\leftrightarrow fa} + k_{fx\leftrightarrow fa}))$ (S1.8)
