# Supplementary material for: Model-assisted analysis of the peach pedicel–fruit system suggests regulation of sugar uptake and a water-saving strategy
Source: J Exp Bot. 2020 May 18;71(12):3463–74. doi: 10.1093/jxb/eraa103 (PMC7307860; doi:10.1093/jxb/eraa103)
Supplement: eraa103_suppl_Supplementary_File002 [file eraa103_suppl_supplementary_file002.pdf]

Table S1.1: Calibrated model parameters and brief descriptions. C: control fruit, G: girdled fruit, 30_94: 30 leaves-to-fruit year 1994, 5_95: 5 leaves-to-fruit year 1995, 30_95 : 30 leaves-to-fruit year 1995; in the fourth column: 10th and 90th percentile of each parameter, computed on the sample composed by the best solutions among all the solutions found by the genetic algorithm. A solution belonged to the best ones if both the $RMSE_C$ and $RMSE_G$ of this solution were up to 5 percent higher than the minimum $RMSE_C$ and $RMSE_G$

Parameter	Description	Value	10th – 90th percentile in the best solutions	Units
$K_{px\leftrightarrow fx}$	Conductance of the water path between the pedicel xylem and the fruit xylem	9.1×10^{-1}	6.8×10^{-1} – 9.1×10^{-1}	(g h^{-1} MPa^{-1})
$K_{pp\leftrightarrow fp}$	Conductance of the water path between the pedicel phloem and the fruit phloem	3.3	1.6 – 3.5	(g h^{-1} MPa^{-1})
$k_{fx\leftrightarrow fa}$<br>$K_{fx\leftrightarrow fa}$*	Conductivity and conductance of the water path between the fruit xylem and the fruit apoplast	1.1×10^{-2}<br>7.4×10^{-1}	6.2×10^{-3} – 1.8×10^{-2}<br>4.4×10^{-1} – 1.3	(g h^{-1} MPa^{-1} cm^{-2})<br>(g h^{-1} MPa^{-1})
$k_{fp\leftrightarrow fa}$<br>$K_{fp\leftrightarrow fa}$*	Conductivity and conductance of the water path between fruit phloem and the fruit apoplast	1.1×10^{-3}<br>7.6×10^{-2}	1.1×10^{-3} – 2.3×10^{-3}<br>7.6×10^{-2} – 1.6×10^{-1}	(g h^{-1} MPa^{-1} cm^{-2})<br>(g h^{-1} MPa^{-1})
$K_{fa\leftrightarrow fs}$<br>$K_{fa\leftrightarrow fs}$*	Conductivity and conductance of the water path between the fruit apoplast and the fruit symplast	9.1×10^{-3}<br>6.4×10^{-1}	8×10^{-3} – 2.3×10^{-2}<br>5.6×10^{-1} – 1.6	(g h^{-1} MPa^{-1} cm^{-2})<br>(g h^{-1} MPa^{-1})
$v_{fp\rightarrow fa}$	Sugar transport rate between the fruit phloem and the fruit apoplast	5.0×10^{-4}	4.6×10^{-4} – 6.6×10^{-4}	(g h^{-1} cm^{-2})
$v_{fa\rightarrow fs}$	Fruit symplast sugar uptake rate	3.6×10^{-2}	3.6×10^{-2} – 8.7×10^{-2}	(h^{-1})
$\Psi_{w,fs}$	Fruit symplast water potential (from 18:00 to 06:00)	-1.7 (C30_94)<br>-1.7 (C30_95)<br>-1.3 (C5_95)<br>-0.89 (G30_94)<br>-1.1 (G30_95)<br>-0.91 (G5_95)	-1.8 – -1.4<br>-1.8 – -1.4<br>-1.3 – -1.1<br>-1.0 – -0.86<br>-1.2 – -1.0<br>-1.0 – -0.87	(MPa)

* conductances are given at the estimated value for the fruits grown at the 5 leaves-to-fruit treatment and at control conditions in 1995, which we took as a reference for the results.
