# Supplementary material for: Model-assisted analysis of the peach pedicel–fruit system suggests regulation of sugar uptake and a water-saving strategy
Source: J Exp Bot. 2020 May 18;71(12):3463–74. doi: 10.1093/jxb/eraa103 (PMC7307860; doi:10.1093/jxb/eraa103)
Supplement: eraa103_suppl_Supplementary_File003 [file eraa103_suppl_supplementary_file003.pdf]

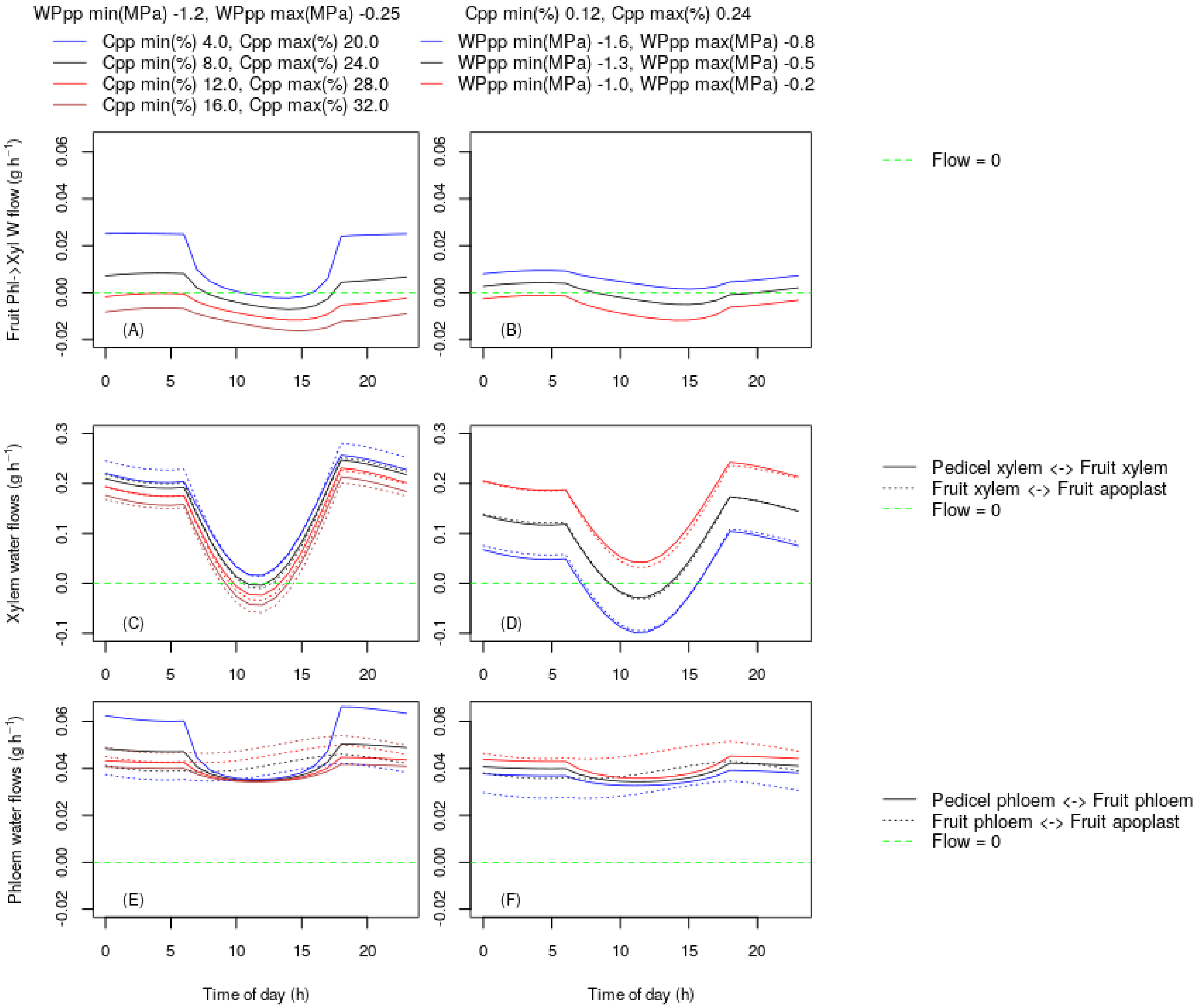

Figure S1.1: Response of the predicted diurnal water flows to different levels of pedicel phloem sugar concentration and pedicel phloem water potential given as input. (A, B): simulated diurnal behavior of the water flow from the fruit phloem to the fruit xylem; (C, D): simulated behavior of the diurnal water flows from the pedicel xylem to the fruit xylem (solid lines) and from the fruit xylem to the fruit apoplast (dotted lines); (E,F) simulated behavior of the diurnal water flows from the pedicel phloem to the fruit phloem (solid lines) and from the fruit phloem to the fruit apoplast (dotted lines). (A, C, E): the pedicel phloem sugar concentration input was set at different levels (low, blue lines; medium, black lines; high, red lines; very high, brown lines), while the pedicel phloem water potential input was set to a fixed diurnal variation; (B, D, F): the pedicel phloem water potential was set at different levels (low – more negative – blue lines; medium, black lines; high – less negative – red lines), while the pedicel phloem sugar concentration was set to a fixed diurnal variation. The dashed green lines correspond to the null values of water flows.

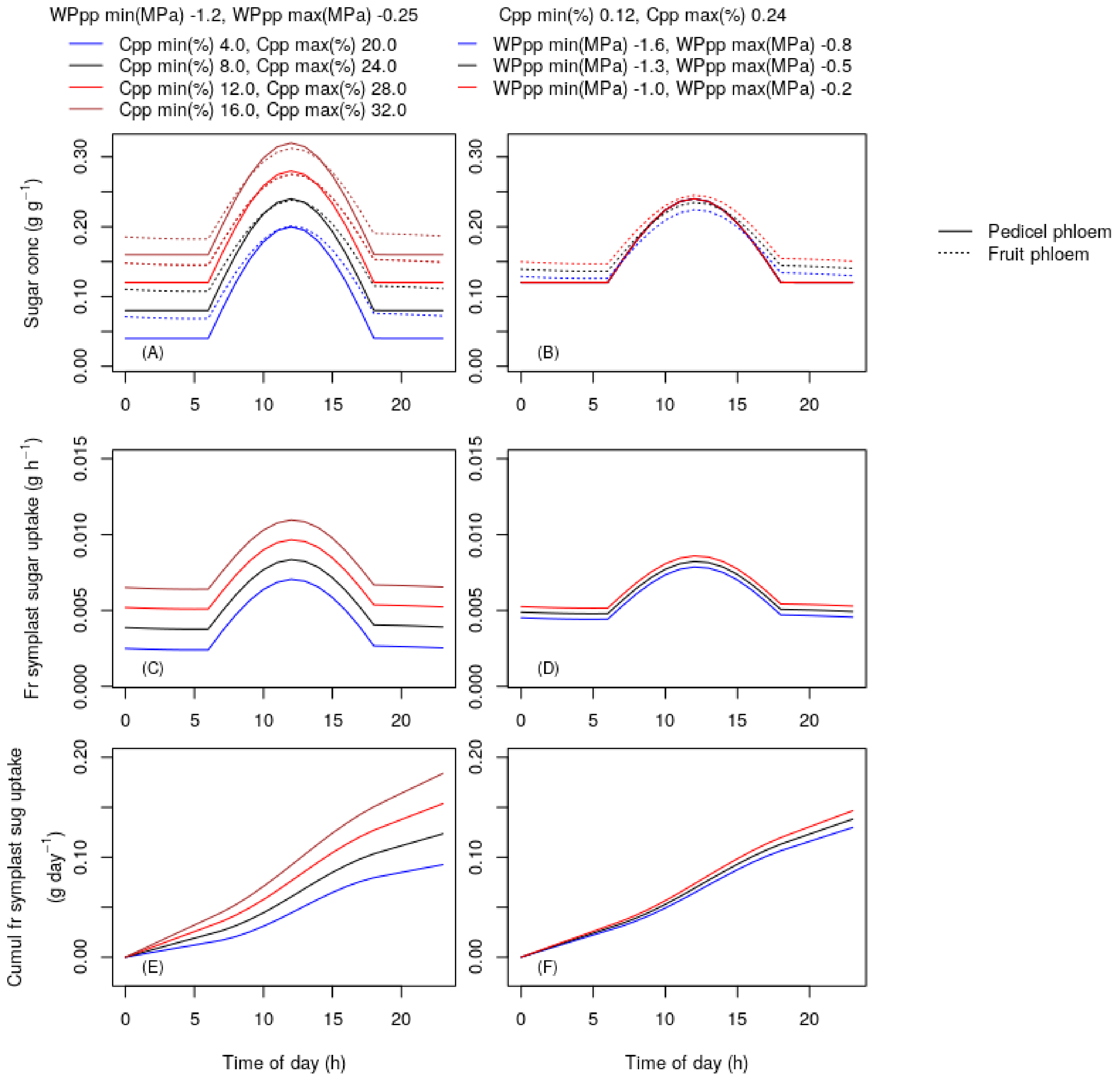

Figure S1.2: Response of the predicted diurnal sugar concentrations and sugar uptake to different levels of pedicel phloem sugar concentration and pedicel phloem water potential given as input. (A, B): simulated diurnal behavior of the pedicel phloem sugar concentrations (input, solid line) and the fruit phloem sugar concentrations (dotted lines). (C, D): simulated behavior of the diurnal fruit symplast sugar uptake. (E, F): simulated behavior of the cumulative fruit symplast sugar uptake. (A, C, E): the pedicel phloem sugar concentration input was set at different levels (low, blue lines; medium, black lines; high, red lines; very high, brown lines), while the pedicel phloem water potential input was set to a fixed diurnal variation; (B, D, F): the pedicel phloem water potential was set at different levels (low – more negative – blue lines; medium, black lines; high – less negative – red lines), while the pedicel phloem sugar concentration was set to a fixed diurnal variation.
